# 3-(2-Chloro-6-fluoro­phen­yl)-1-(2-thien­yl)prop-2-en-1-one

**DOI:** 10.1107/S1600536808024872

**Published:** 2008-08-09

**Authors:** Hoong-Kun Fun, Suchada Chantrapromma, P. S. Patil, S. M. Dharmaprakash

**Affiliations:** a X-ray Crystallography Unit, School of Physics, Universiti Sains Malaysia, 11800 USM, Penang, Malaysia; b Crystal Materials Research Unit, Department of Chemistry, Faculty of Science, Prince of Songkla University, Hat-Yai, Songkhla 90112, Thailand; c Department of Studies in Physics, Mangalore University, Mangalagangotri, Mangalore 574 199, India

## Abstract

The title chalcone derivative, C_13_H_8_ClFOS, crystallized as an inversion twin with two independent mol­ecules in the asymmetric unit. The thio­phene rings in both mol­ecules are disordered over two sites: the ratios of occupancies for the major and minor components in the two mol­ecules are 0.820 (2):0.180 (2) and 0.853 (2):0.147 (2). The dihedral angles between the major and minor components of the thio­phene and benzene rings are 1.13 (18) and 2.2 (6)°, respectively, in one mol­ecule, with corresponding values 6.09 (17) and 1.3 (6)° in the other. Weak intra­molecular C—H⋯O and C—H⋯F inter­actions involving the prop-2-en-1-one group generate an *S*(5)*S*(5) ring motif, whereas a weak intra­molecular C—H⋯Cl contact generates an *S*(6) ring motif. In the crystal structure, mol­ecules of both the major and minor components are linked into infinite one-dimensional chains along the *b* axis. The crystal structure is stabilized by weak C—H⋯O, C—H⋯F, C—H⋯Cl and C—H⋯π inter­actions.

## Related literature

For details of hydrogen-bond motifs, see: Bernstein *et al.* (1995 ▶). For bond-length data, see: Allen *et al.* (1987 ▶). For related structures, see, for example: Fun *et al.* (2008 ▶); Patil *et al.* (2007*b* ▶,*c* ▶). For background to the applications of substituted chalcones, see, for example: Agrinskaya *et al.* (1999 ▶); Chopra *et al.* (2007 ▶). Patil *et al.* (2007*a* ▶).
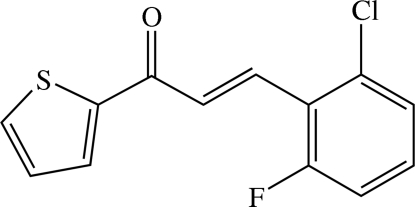


## Experimental

### 

#### Crystal data

C_13_H_8_ClFOS
*M* *_r_* = 266.71
Monoclinic, 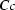

*a* = 12.1137 (3) Å
*b* = 10.5012 (3) Å
*c* = 18.6689 (5) Å
β = 107.882 (3)°
*V* = 2260.11 (11) Å^3^
*Z* = 8
Mo *K*α radiation
μ = 0.51 mm^−1^
*T* = 100.0 (1) K
0.38 × 0.27 × 0.19 mm

#### Data collection

Bruker SMART APEXII CCD area-detector diffractometer
Absorption correction: multi-scan (*SADABS*; Bruker, 2005 ▶) *T*_min_ = 0.830, *T*_max_ = 0.911
26405 measured reflections
6525 independent reflections
5474 reflections with *I* > 2σ(*I*)
*R*_int_ = 0.084

#### Refinement

*R*[*F* ^2^ > 2σ(*F* ^2^)] = 0.063
*wR*(*F* ^2^) = 0.171
*S* = 1.05
6525 reflections
347 parameters
233 restraints
H-atom parameters constrained
Δρ_max_ = 0.83 e Å^−3^
Δρ_min_ = −0.92 e Å^−3^
Absolute structure: Flack (1983 ▶), 3231 Friedel pairs
Flack parameter: 0.43 (7)

### 

Data collection: *APEX2* (Bruker, 2005 ▶); cell refinement: *APEX2*; data reduction: *SAINT* (Bruker, 2005 ▶); program(s) used to solve structure: *SHELXTL* (Sheldrick, 2008 ▶); program(s) used to refine structure: *SHELXTL*; molecular graphics: *SHELXTL*; software used to prepare material for publication: *SHELXTL* and *PLATON* (Spek, 2003 ▶).

## Supplementary Material

Crystal structure: contains datablocks global, I. DOI: 10.1107/S1600536808024872/sj2529sup1.cif

Structure factors: contains datablocks I. DOI: 10.1107/S1600536808024872/sj2529Isup2.hkl

Additional supplementary materials:  crystallographic information; 3D view; checkCIF report

## Figures and Tables

**Table 1:** Hydrogen-bond geometry (Å, °)

*D*—H⋯*A*	*D*—H	H⋯*A*	*D*⋯*A*	*D*—H⋯*A*
C7*A*—H7*AA*⋯F1*A*	0.93	2.39	2.814 (4)	107
C7*A*—H7*AA*⋯O1*A*	0.93	2.45	2.827 (4)	104
C8*A*—H8*AA*⋯Cl1*A*	0.93	2.44	3.103 (3)	129
C7*B*—H7*BA*⋯F1*B*	0.93	2.37	2.794 (3)	107
C7*B*—H7*BA*⋯O1*B*	0.93	2.43	2.812 (3)	104
C8*B*—H8*BA*⋯Cl1*B*	0.93	2.46	3.105 (3)	126
C11*A*—H11*A*⋯F1*A*^i^	0.93	2.54	3.375 (6)	150
C12*A*—H12*A*⋯O1*A*^i^	0.93	2.51	3.402 (5)	161
C12*B*—H12*C*⋯O1*B*^ii^	0.93	2.50	3.427 (4)	174
C3*A*—H3*AA*⋯*Cg*1^iii^	0.93	3.06	3.748 (4)	132
C3*A*—H3*AA*⋯*Cg*3^iii^	0.93	3.14	3.825 (7)	132
C3*B*—H3*BA*⋯*Cg*5^iv^	0.93	3.02	3.778 (4)	140
C11*B*—H11*C*⋯*Cg*6^v^	0.93	2.81	3.677 (4)	155
C13*A*—H13*A*⋯*Cg*2^iv^	0.93	2.82	3.608 (4)	143
C13*A*—H13*A*⋯*Cg*4^iv^	0.93	2.82	3.625 (8)	145
C12*X*—H12*B*⋯*Cg*2^iv^	0.93	3.21	3.835 (16)	126
C12*X*—H12*B*⋯*Cg*4^iv^	0.93	3.18	3.840 (18)	129
C12*Y*—H12*D*⋯*Cg*6^v^	0.93	3.04	3.79 (2)	139

Symmetry codes: (i) 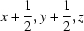; (ii) 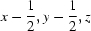; (iii) 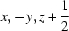; (iv) 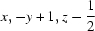; (v) 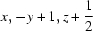. *Cg*1, *Cg*2, *Cg*3, *Cg*4, *Cg*5 and *Cg*6 are the centroids of the S1*A*/C10*A*–C13*A*, S1*B*/C10*B*–C13*B*, S1*X*/C10*A*/C10*X*–C13*X*, S1*Y*/C10*B*/C11*Y*–C13*Y*, C1*A*–C6*A* and C1*B*–C6*B* rings, respectively.

**Fig. 1.**
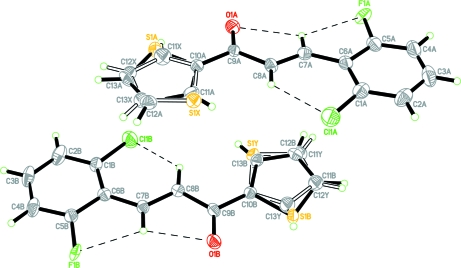
The asymmetric unit of (I), showing 50% probability displacement ellipsoids and the atomic numbering. Weak intramolecular C—H···O, C—H···F and C—H···Cl interactions are drawn as dashed lines. The major disorder components are shown with the solid bonds whereas the minor disorder components are shown with open bonds.

**Fig. 2.**
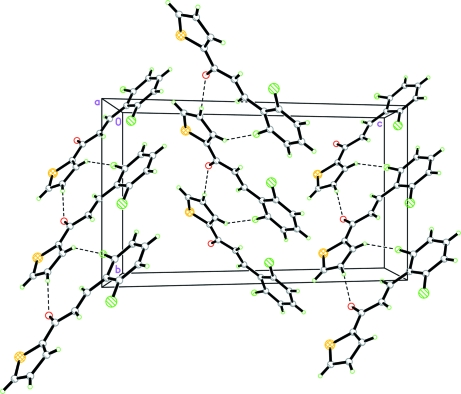
The crystal packing of the major components of (I), viewed along the a axis showing that the molecules are linked in infinite one-dimensional chains approximately along the b axis. Hydrogen bonds are drawn as dashed lines.

## supplementary crystallographic information

### Comment

Chalcone derivatives have received much attention in recent years (Chopra *et al.*, 2007). Some chalcone derivatives have been found to have nonlinear optical properties (Agrinskaya *et al.*, 1999). As part of our research on the synthesis and characterization of chalcone derivatives (Patil *et al.*, 2007*a*, *b*, *c*), we report here the structure of the title compound.

In the asymmetric unit of the title compound (Fig. 1), there are two independent molecules *A* and *B*. The enone fragment, thiophene and benzene rings are individually essentially co-planar. The thiophene rings in both molecules are disordered over two sites which correspond to a rotation of approximately 180° about the single C—C bond (C9—C10). The approximate ratios of occupancies for the major and minor components are 0.820 (2):0.180 (2) in *A* and 0.853 (2):0.147 (2) in *B*. The dihedral angles between the major and and minor components of thiophene and the benzene rings are 1.13 (18)° [S1A/C10A–C13A with C1A–C6A] and 2.2 (6)° [S1X/C10A/C11X–C13X with C1A–C6A] in *A* and 6.09 (17)° [S1B/C10B–C13B with C1B–C6B] and 1.3 (6)° [S1Y/C10B/C11Y–C13Y with C1B–C6B] in *B*. Weak intramolecular C—H···O and C—H···F interactions involving the prop-2-en-1-one moiety generate an S(5)S(5) ring motif whereas a weak intramolecular C—H···Cl interaction generates an S(6) ring motif (Bernstein *et al.*, 1995) (Fig. 1 and Table 1). Bond lengths and angles in molecules *A* and *B* are slightly different but all are in normal ranges (Allen *et al.*, 1987) and comparable to those in a related structure (Fun *et al.*, 2008).

Since the thiophene rings in both molecules are disordered over two sites, there will be four discrete modes of packing in the structure involving the major and minor components. In Fig. 2 only the molecules of the two major components are shown and they are linked into chains along the *b* axis. The crystal is stabilized by weak C—H···O, C—H···F and C—H···Cl interactions (Table 1) and further stabilized by C—H···π interactions (Table 1); *Cg*_1_, *Cg*_2_, *Cg*_3_, *Cg*_4_, *Cg*_5_ and *Cg*_6_ are the centroids of the S1A/C10A–C13A, S1B/C10B—C13B, S1X/C10A/C11X–C13X, S1Y/C10B/C11Y–C13Y, C1A–C6A and C1B–C6B rings, respectively.

### Experimental

The title compound was synthesized by the condensation of 2-chloro-6-fluorobenzaldehyde (0.01 mol, 1.58 g) with 2-acetylthiophene (0.01 mol, 1.07 ml) in methanol (60 ml) in the presence of a catalytic amount of sodium hydroxide solution (5 ml, 30%). After stirring (6 h), the contents of the flask were poured into ice-cold water (500 ml) and left to stand for 5 h. The resulting crude solid was filtered and dried. Colorless single crystals of the title compound suitable for *x*-ray structure determination were grown by slow evaporation of an acetone solution at room temperature.

### Refinement

All H atoms were placed in calculated positions with d(C—H) = 0.93 Å, *U*_iso_=1.2*U*_eq_(C) for CH and aromatic. The highest residual electron density peak is located at 0.36 Å from F1B and the deepest hole is located at 0.56 Å from Cl1B. Similarity and rigid-bond restraints were applied to the disordered atoms in the thiophene rings. Even though the structure contains heavy atoms, the absolute structure cannot be acertained from the Flack parameter because of the racemic twinning of the crystal with BASF = 0.43 (7).

### Crystal data

**Table:**

C_13_H_8_ClFOS	*F*_000_ = 1088
*M**_r_* = 266.71	*D*_x_ = 1.568 Mg m^−^^3^
Monoclinic, *C**c*	Mo *K*α radiation λ = 0.71073 Å
Hall symbol: C -2yc	Cell parameters from 6525 reflections
*a* = 12.1137 (3) Å	θ = 2.3–30.0º
*b* = 10.5012 (3) Å	µ = 0.51 mm^−^^1^
*c* = 18.6689 (5) Å	*T* = 100.0 (1) K
β = 107.882 (3)º	Block, colorless
*V* = 2260.11 (11) Å^3^	0.38 × 0.27 × 0.19 mm
*Z* = 8	

### Data collection

**Table:**

Bruker SMART APEXII CCD area-detector diffractometer	6525 independent reflections
Radiation source: fine-focus sealed tube	5474 reflections with *I* > 2σ(*I*)
Monochromator: graphite	*R*_int_ = 0.084
Detector resolution: 8.33 pixels mm^-1^	θ_max_ = 30.0º
*T* = 100.0(1) K	θ_min_ = 2.3º
ω scans	*h* = −17→17
Absorption correction: multi-scan(SADABS; Bruker, 2005)	*k* = −14→14
*T*_min_ = 0.830, *T*_max_ = 0.911	*l* = −26→26
26405 measured reflections	

### Refinement

**Table:**

Refinement on *F*^2^	Hydrogen site location: inferred from neighbouring sites
Least-squares matrix: full	H-atom parameters constrained
*R*[*F*^2^ > 2σ(*F*^2^)] = 0.063	*w* = 1/[σ^2^(*F*_o_^2^) + (0.089*P*)^2^ + 4.5385*P*] where *P* = (*F*_o_^2^ + 2*F*_c_^2^)/3
*wR*(*F*^2^) = 0.171	(Δ/σ)_max_ < 0.001
*S* = 1.05	Δρ_max_ = 0.83 e Å^−^^3^
6525 reflections	Δρ_min_ = −0.91 e Å^−^^3^
347 parameters	Extinction correction: none
233 restraints	Absolute structure: Flack (1983), 3231 Friedel pairs
Primary atom site location: structure-invariant direct methods	Flack parameter: 0.43 (7)
Secondary atom site location: difference Fourier map	

### Special details

**Table:**

Experimental. The low-temperature data was collected with the Oxford Cyrosystem Cobra low-temperature attachment.
Geometry. All e.s.d.'s (except the e.s.d. in the dihedral angle between two l.s. planes) are estimated using the full covariance matrix. The cell e.s.d.'s are taken into account individually in the estimation of e.s.d.'s in distances, angles and torsion angles; correlations between e.s.d.'s in cell parameters are only used when they are defined by crystal symmetry. An approximate (isotropic) treatment of cell e.s.d.'s is used for estimating e.s.d.'s involving l.s. planes.
Refinement. Refinement of *F*^2^ against ALL reflections. The weighted *R*-factor *wR* and goodness of fit *S* are based on *F*^2^, conventional *R*-factors *R* are based on *F*, with *F* set to zero for negative *F*^2^. The threshold expression of *F*^2^ > σ(*F*^2^) is used only for calculating *R*-factors(gt) *etc*. and is not relevant to the choice of reflections for refinement. *R*-factors based on *F*^2^ are statistically about twice as large as those based on *F*, and *R*- factors based on ALL data will be even larger.

### Fractional atomic coordinates and isotropic or equivalent isotropic displacement parameters (Å^2^)

**Table:**

	*x*	*y*	*z*	*U*_iso_*/*U*_eq_	Occ. (<1)
Cl1A	0.16981 (8)	0.06049 (9)	0.04048 (5)	0.0439 (2)	
F1A	−0.22351 (13)	−0.16353 (19)	−0.01215 (9)	0.0282 (4)	
O1A	−0.23551 (18)	0.1662 (2)	−0.18144 (12)	0.0271 (5)	
C1A	0.0753 (3)	−0.0405 (3)	0.05750 (18)	0.0304 (7)	
C2A	0.1172 (3)	−0.1253 (3)	0.11799 (18)	0.0368 (8)	
H2AA	0.1943	−0.1208	0.1478	0.044*	
C3A	0.0444 (4)	−0.2155 (3)	0.1335 (2)	0.0450 (9)	
H3AA	0.0725	−0.2701	0.1744	0.054*	
C4A	−0.0684 (4)	−0.2251 (3)	0.0895 (2)	0.0408 (9)	
H4AA	−0.1171	−0.2870	0.0989	0.049*	
C5A	−0.1078 (3)	−0.1421 (3)	0.03161 (18)	0.0326 (7)	
C6A	−0.0413 (3)	−0.0465 (3)	0.01129 (16)	0.0248 (6)	
C7A	−0.0979 (3)	0.0341 (3)	−0.05309 (16)	0.0240 (6)	
H7AA	−0.1764	0.0172	−0.0752	0.029*	
C8A	−0.0570 (3)	0.1277 (3)	−0.08580 (17)	0.0250 (6)	
H8AA	0.0204	0.1516	−0.0661	0.030*	
C9A	−0.1314 (2)	0.1940 (2)	−0.15224 (16)	0.0209 (6)	
C10A	−0.0801 (2)	0.2973 (2)	−0.18457 (15)	0.0206 (5)	
S1A	−0.16312 (8)	0.37629 (9)	−0.26092 (5)	0.0256 (2)	0.821 (2)
C11A	0.0339 (4)	0.3415 (5)	−0.1616 (3)	0.0319 (11)	0.821 (2)
H11A	0.0922	0.3078	−0.1212	0.038*	0.821 (2)
C12A	0.0509 (4)	0.4447 (4)	−0.2072 (2)	0.0288 (9)	0.821 (2)
H12A	0.1206	0.4877	−0.1997	0.035*	0.821 (2)
C13A	−0.0498 (3)	0.4716 (3)	−0.2634 (2)	0.0244 (8)	0.821 (2)
H13A	−0.0557	0.5350	−0.2992	0.029*	0.821 (2)
S1X	0.0540 (5)	0.3451 (5)	−0.1503 (3)	0.0244 (12)*	0.179 (2)
C11X	−0.1495 (13)	0.3737 (17)	−0.2410 (11)	0.035 (5)*	0.179 (2)
H11B	−0.2300	0.3692	−0.2597	0.042*	0.179 (2)
C12X	−0.0788 (12)	0.4597 (16)	−0.2659 (9)	0.020 (4)*	0.179 (2)
H12B	−0.1050	0.5093	−0.3091	0.025*	0.179 (2)
C13X	0.0334 (13)	0.4609 (17)	−0.2182 (10)	0.026 (4)*	0.179 (2)
H13B	0.0906	0.5170	−0.2222	0.031*	0.179 (2)
Cl1B	−0.16932 (8)	0.69186 (9)	−0.16305 (5)	0.0416 (2)	
F1B	0.22038 (13)	0.9255 (2)	−0.10610 (9)	0.0291 (4)	
O1B	0.23296 (17)	0.60229 (19)	0.06450 (12)	0.0267 (5)	
C1B	−0.0721 (3)	0.7932 (3)	−0.18114 (17)	0.0250 (6)	
C2B	−0.1171 (3)	0.8706 (4)	−0.24489 (19)	0.0353 (8)	
H2BA	−0.1930	0.8603	−0.2758	0.042*	
C3B	−0.0460 (3)	0.9621 (3)	−0.2606 (2)	0.0352 (7)	
H3BA	−0.0743	1.0140	−0.3026	0.042*	
C4B	0.0651 (3)	0.9774 (3)	−0.21507 (17)	0.0341 (7)	
H4BA	0.1127	1.0397	−0.2254	0.041*	
C5B	0.1055 (3)	0.8987 (3)	−0.15358 (16)	0.0248 (6)	
C6B	0.0415 (2)	0.8020 (3)	−0.13370 (16)	0.0206 (5)	
C7B	0.0981 (2)	0.7253 (2)	−0.06705 (15)	0.0205 (5)	
H7BA	0.1726	0.7514	−0.0401	0.025*	
C8B	0.0591 (3)	0.6225 (3)	−0.03853 (16)	0.0232 (6)	
H8BA	−0.0136	0.5885	−0.0629	0.028*	
C9B	0.1334 (3)	0.5649 (3)	0.03181 (16)	0.0229 (6)	
C10B	0.0835 (2)	0.4603 (3)	0.06389 (15)	0.0195 (5)	
S1B	0.16576 (7)	0.39734 (8)	0.14734 (5)	0.02200 (18)	0.853 (2)
C11B	0.0601 (3)	0.2918 (4)	0.1489 (2)	0.0220 (9)	0.853 (2)
H11C	0.0662	0.2341	0.1877	0.026*	0.853 (2)
C12B	−0.0348 (3)	0.3014 (4)	0.0865 (2)	0.0243 (8)	0.853 (2)
H12C	−0.1007	0.2507	0.0773	0.029*	0.853 (2)
C13B	−0.0199 (4)	0.3982 (4)	0.0380 (2)	0.0222 (8)	0.853 (2)
H13C	−0.0755	0.4179	−0.0074	0.027*	0.853 (2)
S1Y	−0.0509 (5)	0.4042 (6)	0.0261 (3)	0.0182 (12)	0.147 (2)
C11Y	−0.0421 (15)	0.293 (2)	0.0931 (12)	0.028 (5)*	0.147 (2)
H11D	−0.1016	0.2380	0.0947	0.034*	0.147 (2)
C12Y	0.0663 (16)	0.295 (2)	0.1448 (13)	0.028 (6)*	0.147 (2)
H12D	0.0912	0.2418	0.1863	0.033*	0.147 (2)
C13Y	0.1343 (15)	0.3903 (17)	0.1263 (10)	0.028 (5)*	0.147 (2)
H13D	0.2106	0.4045	0.1555	0.034*	0.147 (2)

### Atomic displacement parameters (Å^2^)

**Table:**

	*U*^11^	*U*^22^	*U*^33^	*U*^12^	*U*^13^	*U*^23^
Cl1A	0.0386 (4)	0.0458 (5)	0.0426 (5)	−0.0123 (4)	0.0056 (4)	0.0065 (4)
F1A	0.0116 (6)	0.0517 (10)	0.0204 (7)	−0.0127 (7)	0.0036 (6)	0.0033 (7)
O1A	0.0269 (9)	0.0264 (9)	0.0285 (10)	−0.0043 (8)	0.0090 (8)	0.0000 (8)
C1A	0.0317 (15)	0.0328 (15)	0.0268 (14)	0.0066 (12)	0.0090 (12)	−0.0026 (12)
C2A	0.0403 (17)	0.0429 (17)	0.0254 (15)	0.0147 (15)	0.0074 (14)	−0.0034 (13)
C3A	0.081 (2)	0.0263 (14)	0.0353 (16)	0.0153 (16)	0.0295 (17)	0.0072 (12)
C4A	0.069 (2)	0.0250 (14)	0.0328 (16)	0.0000 (16)	0.0216 (16)	0.0028 (13)
C5A	0.0544 (17)	0.0239 (13)	0.0264 (13)	−0.0046 (13)	0.0225 (12)	−0.0031 (11)
C6A	0.0343 (14)	0.0207 (11)	0.0227 (12)	0.0018 (11)	0.0138 (11)	−0.0031 (9)
C7A	0.0270 (12)	0.0229 (12)	0.0233 (12)	0.0017 (11)	0.0094 (10)	−0.0029 (10)
C8A	0.0279 (13)	0.0218 (12)	0.0264 (13)	−0.0039 (10)	0.0102 (11)	0.0016 (10)
C9A	0.0295 (13)	0.0158 (10)	0.0185 (11)	0.0010 (10)	0.0089 (10)	−0.0008 (9)
C10A	0.0254 (12)	0.0199 (11)	0.0193 (11)	−0.0015 (10)	0.0110 (10)	−0.0031 (9)
S1A	0.0276 (4)	0.0256 (4)	0.0234 (4)	−0.0015 (3)	0.0078 (3)	0.0045 (3)
C11A	0.0287 (19)	0.044 (2)	0.0208 (18)	−0.0121 (17)	0.0050 (15)	−0.0007 (15)
C12A	0.0340 (17)	0.0274 (17)	0.0274 (18)	−0.0059 (15)	0.0130 (14)	−0.0062 (14)
C13A	0.0245 (15)	0.0188 (14)	0.0354 (17)	−0.0091 (13)	0.0172 (13)	−0.0001 (12)
Cl1B	0.0370 (4)	0.0403 (4)	0.0428 (4)	−0.0098 (3)	0.0053 (4)	0.0077 (4)
F1B	0.0074 (6)	0.0587 (11)	0.0163 (7)	−0.0102 (7)	−0.0037 (5)	0.0189 (7)
O1B	0.0227 (9)	0.0250 (9)	0.0307 (10)	−0.0037 (8)	0.0059 (8)	0.0044 (8)
C1B	0.0251 (13)	0.0275 (13)	0.0227 (13)	0.0006 (11)	0.0076 (11)	0.0039 (11)
C2B	0.0312 (15)	0.0475 (18)	0.0287 (15)	0.0180 (14)	0.0111 (12)	0.0114 (13)
C3B	0.0498 (17)	0.0322 (14)	0.0287 (14)	0.0198 (14)	0.0196 (13)	0.0133 (12)
C4B	0.0592 (19)	0.0254 (13)	0.0247 (14)	0.0095 (13)	0.0235 (13)	0.0042 (11)
C5B	0.0328 (14)	0.0222 (12)	0.0212 (12)	−0.0005 (11)	0.0108 (11)	−0.0018 (10)
C6B	0.0231 (12)	0.0189 (11)	0.0222 (12)	0.0012 (9)	0.0108 (10)	−0.0025 (9)
C7B	0.0229 (11)	0.0195 (11)	0.0210 (12)	0.0001 (10)	0.0096 (10)	0.0004 (9)
C8B	0.0256 (13)	0.0210 (12)	0.0220 (12)	−0.0015 (10)	0.0059 (10)	−0.0003 (10)
C9B	0.0278 (13)	0.0172 (11)	0.0253 (13)	−0.0019 (10)	0.0108 (11)	−0.0005 (10)
C10B	0.0208 (11)	0.0191 (11)	0.0187 (11)	0.0013 (10)	0.0061 (9)	−0.0008 (9)
S1B	0.0200 (4)	0.0232 (3)	0.0219 (4)	−0.0004 (3)	0.0051 (3)	0.0069 (3)
C11B	0.0279 (15)	0.0198 (15)	0.0219 (15)	0.0022 (12)	0.0132 (12)	0.0063 (11)
C12B	0.0255 (15)	0.0203 (14)	0.0292 (17)	−0.0052 (12)	0.0117 (13)	−0.0045 (12)
C13B	0.0195 (16)	0.0220 (15)	0.0212 (16)	0.0032 (13)	0.0005 (13)	0.0009 (12)
S1Y	0.006 (2)	0.029 (2)	0.015 (2)	−0.0037 (18)	−0.0044 (16)	0.0052 (18)

### Geometric parameters (Å, °)

**Table:**

Cl1A—C1A	1.661 (4)	Cl1B—C1B	1.697 (3)
F1A—C5A	1.407 (4)	F1B—C5B	1.430 (3)
O1A—C9A	1.245 (3)	O1B—C9B	1.237 (3)
C1A—C2A	1.405 (5)	C1B—C6B	1.392 (4)
C1A—C6A	1.413 (4)	C1B—C2B	1.405 (4)
C2A—C3A	1.385 (6)	C2B—C3B	1.380 (5)
C2A—H2AA	0.9300	C2B—H2BA	0.9300
C3A—C4A	1.365 (6)	C3B—C4B	1.363 (5)
C3A—H3AA	0.9300	C3B—H3BA	0.9300
C4A—C5A	1.355 (5)	C4B—C5B	1.377 (4)
C4A—H4AA	0.9300	C4B—H4BA	0.9300
C5A—C6A	1.410 (4)	C5B—C6B	1.395 (4)
C6A—C7A	1.458 (4)	C6B—C7B	1.464 (4)
C7A—C8A	1.331 (4)	C7B—C8B	1.351 (4)
C7A—H7AA	0.9300	C7B—H7BA	0.9300
C8A—C9A	1.466 (4)	C8B—C9B	1.474 (4)
C8A—H8AA	0.9300	C8B—H8BA	0.9300
C9A—C10A	1.470 (4)	C9B—C10B	1.467 (4)
C10A—C11X	1.384 (14)	C10B—C13Y	1.355 (14)
C10A—C11A	1.394 (5)	C10B—C13B	1.363 (5)
C10A—S1X	1.631 (6)	C10B—S1Y	1.671 (6)
C10A—S1A	1.688 (3)	C10B—S1B	1.704 (3)
S1A—C13A	1.712 (4)	S1B—C11B	1.700 (4)
C11A—C12A	1.431 (6)	C11B—C12B	1.366 (5)
C11A—H11A	0.9300	C11B—H11C	0.9300
C12A—C13A	1.372 (5)	C12B—C13B	1.409 (6)
C12A—H12A	0.9300	C12B—H12C	0.9300
C13A—H13A	0.9300	C13B—H13C	0.9300
S1X—C13X	1.718 (14)	S1Y—C11Y	1.692 (16)
C11X—C12X	1.418 (16)	C11Y—C12Y	1.369 (16)
C11X—H11B	0.9300	C11Y—H11D	0.9300
C12X—C13X	1.377 (15)	C12Y—C13Y	1.402 (17)
C12X—H12B	0.9300	C12Y—H12D	0.9300
C13X—H13B	0.9300	C13Y—H13D	0.9300
			
C2A—C1A—C6A	120.6 (3)	C6B—C1B—C2B	123.5 (3)
C2A—C1A—Cl1A	117.3 (3)	C6B—C1B—Cl1B	121.7 (2)
C6A—C1A—Cl1A	122.0 (2)	C2B—C1B—Cl1B	114.7 (2)
C3A—C2A—C1A	120.4 (3)	C3B—C2B—C1B	118.5 (3)
C3A—C2A—H2AA	119.8	C3B—C2B—H2BA	120.8
C1A—C2A—H2AA	119.8	C1B—C2B—H2BA	120.8
C4A—C3A—C2A	120.5 (3)	C4B—C3B—C2B	120.7 (3)
C4A—C3A—H3AA	119.7	C4B—C3B—H3BA	119.6
C2A—C3A—H3AA	119.7	C2B—C3B—H3BA	119.6
C5A—C4A—C3A	118.4 (4)	C3B—C4B—C5B	118.6 (3)
C5A—C4A—H4AA	120.8	C3B—C4B—H4BA	120.7
C3A—C4A—H4AA	120.8	C5B—C4B—H4BA	120.7
C4A—C5A—F1A	113.8 (3)	C4B—C5B—C6B	125.1 (3)
C4A—C5A—C6A	125.6 (3)	C4B—C5B—F1B	115.3 (3)
F1A—C5A—C6A	120.5 (3)	C6B—C5B—F1B	119.5 (2)
C5A—C6A—C1A	114.5 (3)	C1B—C6B—C5B	113.6 (3)
C5A—C6A—C7A	118.2 (3)	C1B—C6B—C7B	128.1 (3)
C1A—C6A—C7A	127.4 (3)	C5B—C6B—C7B	118.3 (2)
C8A—C7A—C6A	131.3 (3)	C8B—C7B—C6B	130.2 (3)
C8A—C7A—H7AA	114.4	C8B—C7B—H7BA	114.9
C6A—C7A—H7AA	114.4	C6B—C7B—H7BA	114.9
C7A—C8A—C9A	121.3 (3)	C7B—C8B—C9B	119.2 (3)
C7A—C8A—H8AA	119.4	C7B—C8B—H8BA	120.4
C9A—C8A—H8AA	119.4	C9B—C8B—H8BA	120.4
O1A—C9A—C8A	122.5 (3)	O1B—C9B—C10B	119.8 (3)
O1A—C9A—C10A	119.5 (2)	O1B—C9B—C8B	123.0 (3)
C8A—C9A—C10A	118.0 (2)	C10B—C9B—C8B	117.1 (2)
C11X—C10A—C11A	110.8 (7)	C13Y—C10B—C13B	99.8 (8)
C11X—C10A—C9A	120.4 (7)	C13Y—C10B—C9B	128.5 (8)
C11A—C10A—C9A	128.6 (3)	C13B—C10B—C9B	131.5 (3)
C11X—C10A—S1X	114.8 (7)	C13Y—C10B—S1Y	107.3 (8)
C9A—C10A—S1X	124.2 (3)	C9B—C10B—S1Y	124.2 (3)
C11A—C10A—S1A	111.9 (3)	C13B—C10B—S1B	110.7 (3)
C9A—C10A—S1A	119.5 (2)	C9B—C10B—S1B	117.7 (2)
S1X—C10A—S1A	116.3 (3)	S1Y—C10B—S1B	118.0 (3)
C10A—S1A—C13A	92.04 (16)	C11B—S1B—C10B	92.09 (15)
C10A—C11A—C12A	112.2 (4)	C12B—C11B—S1B	112.3 (3)
C10A—C11A—H11A	123.9	C12B—C11B—H11C	123.9
C12A—C11A—H11A	123.9	S1B—C11B—H11C	123.9
C13A—C12A—C11A	110.8 (4)	C11B—C12B—C13B	111.1 (3)
C13A—C12A—H12A	124.6	C11B—C12B—H12C	124.4
C11A—C12A—H12A	124.6	C13B—C12B—H12C	124.4
C12A—C13A—S1A	112.9 (3)	C10B—C13B—C12B	113.8 (3)
C12A—C13A—H13A	123.5	C10B—C13B—H13C	123.1
S1A—C13A—H13A	123.5	C12B—C13B—H13C	123.1
C10A—S1X—C13X	91.6 (6)	C10B—S1Y—C11Y	95.7 (6)
C10A—C11X—C12X	109.3 (11)	C12Y—C11Y—S1Y	109.6 (13)
C10A—C11X—H11B	125.3	C12Y—C11Y—H11D	125.2
C12X—C11X—H11B	125.3	S1Y—C11Y—H11D	125.2
C13X—C12X—C11X	111.7 (13)	C11Y—C12Y—C13Y	110.5 (15)
C13X—C12X—H12B	124.1	C11Y—C12Y—H12D	124.7
C11X—C12X—H12B	124.1	C13Y—C12Y—H12D	124.7
C12X—C13X—S1X	111.1 (11)	C10B—C13Y—C12Y	116.8 (13)
C12X—C13X—H13B	124.4	C10B—C13Y—H13D	121.6
S1X—C13X—H13B	124.4	C12Y—C13Y—H13D	121.6
			
C6A—C1A—C2A—C3A	−0.5 (5)	C6B—C1B—C2B—C3B	−1.5 (5)
Cl1A—C1A—C2A—C3A	−177.4 (3)	Cl1B—C1B—C2B—C3B	175.4 (3)
C1A—C2A—C3A—C4A	1.3 (5)	C1B—C2B—C3B—C4B	−0.2 (5)
C2A—C3A—C4A—C5A	−1.7 (5)	C2B—C3B—C4B—C5B	0.7 (5)
C3A—C4A—C5A—F1A	177.4 (3)	C3B—C4B—C5B—C6B	0.6 (5)
C3A—C4A—C5A—C6A	1.4 (5)	C3B—C4B—C5B—F1B	−176.4 (3)
C4A—C5A—C6A—C1A	−0.5 (5)	C2B—C1B—C6B—C5B	2.5 (4)
F1A—C5A—C6A—C1A	−176.3 (3)	Cl1B—C1B—C6B—C5B	−174.2 (2)
C4A—C5A—C6A—C7A	179.7 (3)	C2B—C1B—C6B—C7B	−178.7 (3)
F1A—C5A—C6A—C7A	3.9 (4)	Cl1B—C1B—C6B—C7B	4.6 (4)
C2A—C1A—C6A—C5A	0.1 (4)	C4B—C5B—C6B—C1B	−2.1 (4)
Cl1A—C1A—C6A—C5A	176.8 (2)	F1B—C5B—C6B—C1B	174.8 (3)
C2A—C1A—C6A—C7A	179.8 (3)	C4B—C5B—C6B—C7B	179.0 (3)
Cl1A—C1A—C6A—C7A	−3.4 (5)	F1B—C5B—C6B—C7B	−4.1 (4)
C5A—C6A—C7A—C8A	−177.9 (3)	C1B—C6B—C7B—C8B	5.9 (5)
C1A—C6A—C7A—C8A	2.3 (5)	C5B—C6B—C7B—C8B	−175.4 (3)
C6A—C7A—C8A—C9A	178.5 (3)	C6B—C7B—C8B—C9B	−178.0 (3)
C7A—C8A—C9A—O1A	−0.3 (4)	C7B—C8B—C9B—O1B	−3.7 (4)
C7A—C8A—C9A—C10A	179.3 (3)	C7B—C8B—C9B—C10B	175.0 (3)
O1A—C9A—C10A—C11X	8.0 (11)	O1B—C9B—C10B—C13Y	−3.0 (13)
C8A—C9A—C10A—C11X	−171.6 (11)	C8B—C9B—C10B—C13Y	178.2 (12)
O1A—C9A—C10A—C11A	−178.8 (4)	O1B—C9B—C10B—C13B	−176.5 (4)
C8A—C9A—C10A—C11A	1.6 (5)	C8B—C9B—C10B—C13B	4.7 (5)
O1A—C9A—C10A—S1X	178.4 (3)	O1B—C9B—C10B—S1Y	178.4 (4)
C8A—C9A—C10A—S1X	−1.2 (4)	C8B—C9B—C10B—S1Y	−0.4 (5)
O1A—C9A—C10A—S1A	−0.2 (4)	O1B—C9B—C10B—S1B	1.6 (4)
C8A—C9A—C10A—S1A	−179.8 (2)	C8B—C9B—C10B—S1B	−177.1 (2)
C11X—C10A—S1A—C13A	81 (5)	C13Y—C10B—S1B—C11B	−19 (5)
C11A—C10A—S1A—C13A	−1.0 (3)	C13B—C10B—S1B—C11B	−1.8 (3)
C9A—C10A—S1A—C13A	−179.7 (2)	C9B—C10B—S1B—C11B	179.7 (3)
S1X—C10A—S1A—C13A	1.6 (3)	S1Y—C10B—S1B—C11B	2.7 (3)
C11X—C10A—C11A—C12A	−6.0 (11)	C10B—S1B—C11B—C12B	1.5 (3)
C9A—C10A—C11A—C12A	−179.8 (3)	S1B—C11B—C12B—C13B	−0.9 (5)
S1X—C10A—C11A—C12A	−151 (5)	C13Y—C10B—C13B—C12B	5.0 (11)
S1A—C10A—C11A—C12A	1.6 (5)	C9B—C10B—C13B—C12B	179.9 (3)
C10A—C11A—C12A—C13A	−1.5 (6)	S1Y—C10B—C13B—C12B	−150 (3)
C11A—C12A—C13A—S1A	0.8 (5)	S1B—C10B—C13B—C12B	1.6 (4)
C10A—S1A—C13A—C12A	0.1 (3)	C11B—C12B—C13B—C10B	−0.5 (5)
C11X—C10A—S1X—C13X	−7.8 (13)	C13Y—C10B—S1Y—C11Y	1.6 (14)
C11A—C10A—S1X—C13X	29 (4)	C13B—C10B—S1Y—C11Y	28 (2)
C9A—C10A—S1X—C13X	−178.6 (8)	C9B—C10B—S1Y—C11Y	−179.5 (10)
S1A—C10A—S1X—C13X	0.0 (8)	S1B—C10B—S1Y—C11Y	−2.8 (10)
C11A—C10A—C11X—C12X	9.6 (18)	C10B—S1Y—C11Y—C12Y	−1(2)
C9A—C10A—C11X—C12X	−176.1 (11)	S1Y—C11Y—C12Y—C13Y	1(3)
S1X—C10A—C11X—C12X	12.7 (19)	C13B—C10B—C13Y—C12Y	−5(2)
S1A—C10A—C11X—C12X	−91 (6)	C9B—C10B—C13Y—C12Y	179.7 (16)
C10A—C11X—C12X—C13X	−12 (2)	S1Y—C10B—C13Y—C12Y	−1(2)
C11X—C12X—C13X—S1X	7(2)	S1B—C10B—C13Y—C12Y	159 (6)
C10A—S1X—C13X—C12X	0.4 (16)	C11Y—C12Y—C13Y—C10B	0(3)

### Hydrogen-bond geometry (Å, °)

**Table:**

*D*—H···*A*	*D*—H	H···*A*	*D*···*A*	*D*—H···*A*
C7A—H7AA···F1A	0.93	2.39	2.814 (4)	107
C7A—H7AA···O1A	0.93	2.45	2.827 (4)	104
C8A—H8AA···Cl1A	0.93	2.44	3.103 (3)	129
C7B—H7BA···F1B	0.93	2.37	2.794 (3)	107
C7B—H7BA···O1B	0.93	2.43	2.812 (3)	104
C8B—H8BA···Cl1B	0.93	2.46	3.105 (3)	126
C11A—H11A···F1A^i^	0.93	2.54	3.375 (6)	150
C12A—H12A···O1A^i^	0.93	2.51	3.402 (5)	161
C12B—H12C···O1B^ii^	0.93	2.50	3.427 (4)	174
C3A—H3AA···Cg1^iii^	0.93	3.06	3.748 (4)	132
C3A—H3AA···Cg3^iii^	0.93	3.14	3.825 (7)	132
C3B—H3BA···Cg5^iv^	0.93	3.02	3.778 (4)	140
C11B—H11C···Cg6^v^	0.93	2.81	3.677 (4)	155
C13A—H13A···Cg2^iv^	0.93	2.82	3.608 (4)	143
C13A—H13A···Cg4^iv^	0.93	2.82	3.625 (8)	145
C12X—H12B···Cg2^iv^	0.93	3.21	3.835 (16)	126
C12X—H12B···Cg4^iv^	0.93	3.18	3.840 (18)	129
C12Y—H12D···Cg6^v^	0.93	3.04	3.79 (2)	139

Symmetry codes:  (i) *x*+1/2, *y*+1/2, *z*; (ii) *x*−1/2, *y*−1/2, *z*; (iii) *x*, −*y*, *z*+1/2; (iv) *x*, −*y*+1, *z*−1/2; (v) *x*, −*y*+1, *z*+1/2.

## References

[bb1] Agrinskaya, N. V., Lukoshkin, V. A., Kudryavtsev, V. V., Nosova, G. I., Solovskaya, N. A. & Yakimanski, A. V. (1999). *Phys. Solid State* **41**, 1914–1917.

[bb2] Allen, F. H., Kennard, O., Watson, D. G., Brammer, L., Orpen, A. G. & Taylor, R. (1987). *J. Chem. Soc. Perkin Trans. 2*, pp. S1–S19.

[bb3] Bernstein, J., Davis, R. E., Shimoni, L. & Chang, N.-L. (1995). *Angew. Chem. Int. Ed. Engl* **34**, 1555–1573.

[bb4] Bruker (2005). *APEX2*, *SAINT* and *SADABS* Bruker AXS Inc., Madison, Wisconsin, USA.

[bb5] Chopra, D., Mohan, T. P., Vishalakshi, B. & Guru Row, T. N. (2007). *Acta Cryst.* C**63**, o704–o710. 10.1107/S0108270107049426 18057618

[bb6] Flack, H. D. (1983). *Acta Cryst.* A**39**, 876–881.

[bb7] Fun, H.-K., Jebas, S. R., Patil, P. S. & Dharmaprakash, S. M. (2008). *Acta Cryst.* E**64**, o1510–o1511. 10.1107/S1600536808021375 PMC2962137 21203219

[bb8] Patil, P. S., Dharmaprakash, S. M., Ramakrishna, K., Fun, H.-K., Sai Santosh Kumar, R. & Rao, D. N. (2007*a*). *J. Cryst. Growth* **303**, 520–524.

[bb9] Patil, P. S., Fun, H.-K., Chantrapromma, S. & Dharmaprakash, S. M. (2007*b*). *Acta Cryst.* E**63**, o2497–o2498.

[bb10] Patil, P. S., Teh, J. B.-J., Fun, H.-K., Razak, I. A. & Dharmaprakash, S. M. (2007*c*). *Acta Cryst.* E**63**, o2122–o2123.

[bb11] Sheldrick, G. M. (2008). *Acta Cryst.* A**64**, 112–122. 10.1107/S0108767307043930 18156677

[bb12] Spek, A. L. (2003). *J. Appl. Cryst.* **36**, 7–13.

